# The human cortex possesses a reconfigurable dynamic network architecture that is disrupted in psychosis

**DOI:** 10.1038/s41467-018-03462-y

**Published:** 2018-03-20

**Authors:** Jenna M. Reinen, Oliver Y. Chén, R. Matthew Hutchison, B. T. Thomas Yeo, Kevin M. Anderson, Mert R. Sabuncu, Dost Öngür, Joshua L. Roffman, Jordan W. Smoller, Justin T. Baker, Avram J. Holmes

**Affiliations:** 1 Department of Psychology, Yale University, New Haven, CT 06520 USA; 2 Department of Psychology, Harvard University, Cambridge, MA 02138 USA; 3 Department of Electrical & Computer Engineering, Clinical Imaging Research Centre, Singapore Institute for Neurotechnology & Memory Network Programme, National University of Singapore, Singapore, 117583 Singapore; 4 Athinoula A. Martinos Center for Biomedical Imaging, Massachusetts General Hospital, Harvard Medical School, Charlestown, MA 02129 USA; 5 School of Electrical and Computer Engineering and Meinig School of Biomedical Engineering, Cornell University, Ithaca, NY 14853 USA; 6 Department of Psychiatry, Psychotic Disorders Division, McLean Hospital, Belmont, MA 02478 USA; 7 Department of Psychiatry, Massachusetts General Hospital, Harvard Medical School, Boston, MA 02114 USA; 8 Psychiatric Neuroimaging Research Division, Massachusetts General Hospital, Charlestown, MA 02129 USA; 9 Department of Psychiatry, Yale University, New Haven, CT 06511 USA

## Abstract

Higher-order cognition emerges through the flexible interactions of large-scale brain networks, an aspect of temporal coordination that may be impaired in psychosis. Here, we map the dynamic functional architecture of the cerebral cortex in healthy young adults, leveraging this atlas of transient network configurations (states), to identify state- and network-specific disruptions in patients with schizophrenia and psychotic bipolar disorder. We demonstrate that dynamic connectivity profiles are reliable within participants, and can act as a fingerprint, identifying specific individuals within a larger group. Patients with psychotic illness exhibit intermittent disruptions within cortical networks previously associated with the disease, and the individual connectivity profiles within specific brain states predict the presence of active psychotic symptoms. Taken together, these results provide evidence for a reconfigurable dynamic architecture in the general population and suggest that prior reports of network disruptions in psychosis may reflect symptom-relevant transient abnormalities, rather than a time-invariant global deficit.

## Abstract (web summary)

Temporal changes in brain dynamics are linked with cognitive abilities, but neither their stability nor relationship to psychosis is clear. Here, authors describe the dynamic neural architecture in healthy controls and patients with psychosis and find that they are stable over time and can predict psychotic symptoms.

## Introduction

The human cortex is organized into large-scale networks with complex patterns of functional coupling^1–3^. While substantial progress has been made delineating aspects of this intricate architecture, research in this domain has traditionally relied on static analytic approaches that assume stable patterns of connectivity across time^4^. However, the brain is not a static organ, and time-varying profiles of network connectivity are evident across a broad range of task states^5,6^ and during periods of unconstrained rest^7–9^, (but see ^10,11^). Variability in the expression of dynamic brain states links to cognition^12–15^, learning, and the presence of psychiatric illnesses like schizophrenia^16–18^ that are characterized by a breakdown in cortical information processing. Despite the importance of understanding the relations that link temporal descriptions of brain function with behavior, the core features of dynamic network organization, the stability of individually specific signatures of time-resolved connectivity, and their associated relevance to the disease, remain unclear.

Patterns of spontaneous brain activity and connectivity have been a focused topic of study in electrophysiological recordings at the level of cells, local fields, and surface electroencephalograms (EEGs)^8^. While formal biophysical models linking the activity of neuronal populations with large-scale brain systems have yet to be established^19^, features of oscillatory neural activity, such as those observed at high temporal resolutions, may be reflected in hemodynamic fluctuations^20^. Transient quasi-stable patterns detected in EEGs (microstates), for example, spatially correlate with network patterns observed through intrinsic functional connectivity magnetic resonance imaging^21–23^. Although macro-scale network dynamics have been linked with changes in arousal^13^, attention^14,15^, and autonomic activity^24^, the biological bases and behavioral significance of these spontaneous fluctuations remain unresolved. There are at least two reasons for this lack of consensus. First, prior studies of functional network dynamics have largely focused on single clustering solutions in isolation, choosing a fixed number of temporal states a priori. As a result, a functional atlas of transient network configurations, or brain states, that are present throughout the population has not been fully characterized. Second, analyses of transient network function have principally focused on establishing the existence of a general architecture of dynamic connectivity shared across the population. Static patterns of intrinsic connectivity are heritable^25,26^ and act as a trait-like fingerprint that can accurately identify specific people from a large group^27–29^. There is a reason to believe that a substantial portion of the dynamic connectome may be unique to each individual. Despite the importance of establishing if time-resolved network function acts as an individual specific signature, the extent to which dynamic connectivity profiles possess intra-subject reliability and capture inter-subject variability has yet to be determined.

Although time-resolved analyses of network organization have largely focused on the study of healthy populations, there is preliminary evidence to suggest that network dysfunction in psychosis may emerge through alterations in the core dynamic architecture of the brain^16–18^. Psychotic illnesses (including schizophrenia, schizoaffective disorder, and bipolar disorder with psychotic features) are marked by broad disruptions across cortical association networks, potentially contributing to widespread changes in information processing^30–34^. By one view, impaired network connectivity in patient populations might be time-invariant, emerging through stable deficits in brain function. An alternate possibility is that aspects of the functional impairments observed in psychosis reflect transient abnormalities preferentially evident during the expression of particular network configurations^17,35^. Converging evidence suggests that aberrant oscillatory activity may link to core symptoms of schizophrenia, including the presence of hallucinations^35^. Patients with schizophrenia exhibit reduced dynamism, spending longer periods of time within single brain states and demonstrating a less-variable repertoire of dynamic network configurations^36^. Relative to healthy populations, patients with schizophrenia dwell more in network configurations typified by reduced large-scale connectivity, while also showing muted cross-network negative correlations, for instance, between default and other networks^18^. Dynamic analyses of network function may distinguish clinical groups, providing information that is inaccessible through static connectivity analysis^17^. The incorporation of time-resolved analyses of network function could provide a more sensitive or specific marker of the disease than static approaches, potentially associating with the illness course and/or the presence of distinct symptom profiles. As impairments in attention, learning, and executive functioning are common across neuropsychiatric disorders^32^, research in this domain could provide novel insights into the biology of illness as we work to predict the onset, track disease states, and optimize treatment response.

Here, we applied a sliding-window approach to characterize the reconfigurable architecture of large-scale brain networks in a sample of healthy young adults and individuals with psychotic illness^37^. Among healthy adults, we demonstrate that the resulting dynamic connectivity profiles are reliably expressed across scans and visits, acting as a biological signature that can identify specific individuals within a larger group. Patients with psychotic illness exhibited intermittent disruptions within cortical association networks previously associated with the disease. Individual connectivity profiles within specific brain states predicted the presence of active psychotic symptoms, operationalized as meeting clinician-rated symptomatic diagnostic criteria with the presence of delusions and/or hallucinations in the past month^38^. This property of dynamic network function generalized to a held-out sample of patients. These collective results suggest a key role for functional network dynamics in human cognition, and highlight how specific breakdowns in time-varying profiles of network connectivity may link with the presence of distinct symptom profiles in psychiatric illnesses.

## Results

### Core dynamic network configurations

**Fig. 1:**
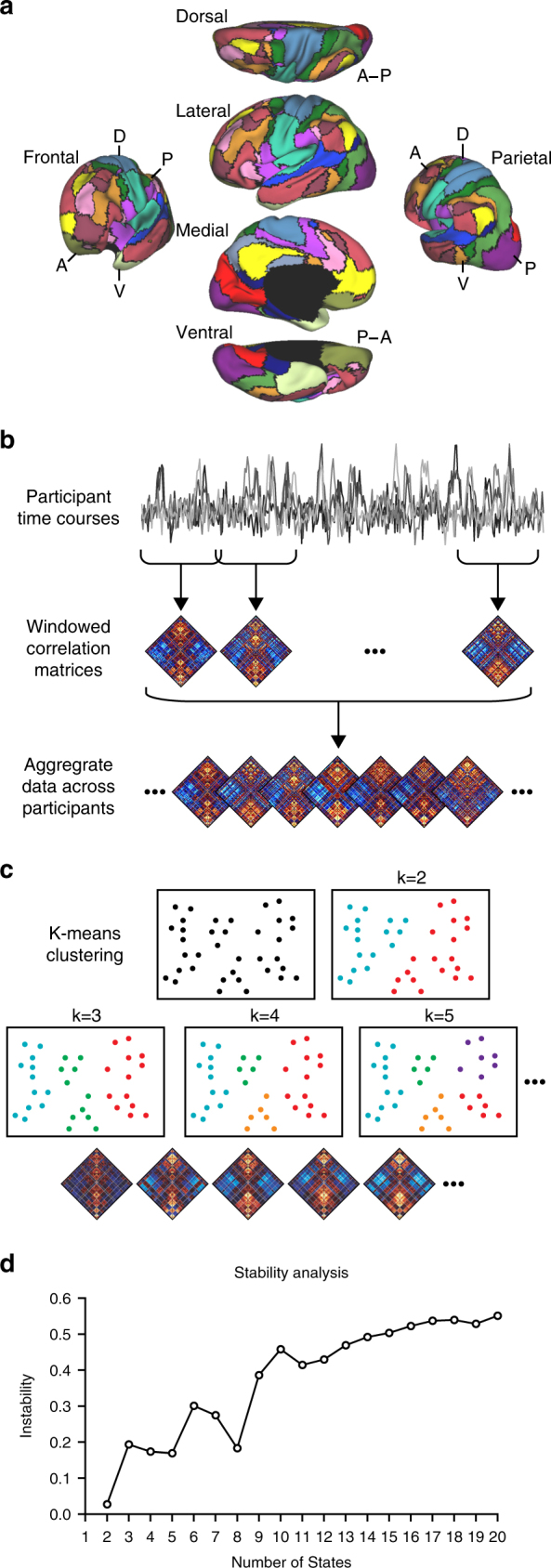
Detecting multiple functional connectivity states using a sliding-window approach. **a** The functional network organization of the human cerebral cortex is revealed through intrinsic functional connectivity. Colors reflect regions estimated to be within the same network determined based on the 17-network solution from Yeo et al.^39^. The map is displayed for multiple views of the left hemisphere in Caret PALS space^79^. **b** Correlation matrices are computed across regions from windowed portions (width = 33 s) of each participant’s component time series (*n* = 1919), and aggregated across the full sample. **c** K-means clustering was applied to identify repeated patterns of connectivity (brain states). **d** Instability of the clustering algorithm is plotted as a function of the number of estimated states (2–20). As expected based on increasing solution space (complexity), instability was greater with increasing the number of estimated states per solution. The local minima of the graphs observable at the 2-, 4-, 5-, and 8-state solutions indicate the number of states that can be stably estimated by the selected clustering algorithm with the present data. Resampling over time (sliding windows) and across space (regions of interest) yields comparable results (see Supplementary Figure 1). In this article, we focus on state solutions 2 through 8 to provide a broad survey of the solution space

The human brain can exhibit a multitude of possible transient connectivity patterns comprised of varying network configurations. As an initial step in understanding temporal shifts in this dynamic architecture over time, we aimed to identify a core or canonical set of transient brain states conserved across individuals. First, we coupled a population atlas of large-scale cortical networks^39^ and a sliding-window approach (11 time points; width = 33 s^7,8,40–42^) to estimate time-varying connectivity profiles within resting-state scans from 1919 healthy young adults (Fig. 1; see Methods for information on data acquisition and preparation)^43^. These data were collapsed across participants before we applied k-means clustering to estimate solutions yielding from 2 to 20 brain states. Note that the brain states defined through k-means clustering do not necessarily have sharp boundaries, cleanly separating them from other network configurations. Rather, k-means clustering identifies sets of time-varying network configurations with common features, grouping them into clusters that are more similar to each other than to configurations in other clusters. The associated groupings can vary across clustering solutions as their complexity increases, revealing intermediate network configurations between more geographically separated states. To estimate the viability of the resulting solutions, we analyzed the population-level consistency of each clustering algorithm^39,44,45^. Consistent with an expansion in the solution space, the clustering became less stable as the number of estimated brain states increased (Fig. 1d and Supplementary Figure 1). Analyses indicated relative stability in state solutions 2–8, with 2-, 4-, 5-, and 8-state solutions displaying points of increased stability. The stable nature of the observed state solutions was robust to changes in data quality (see Methods, Noise constraints). As such, our analyses going forward focus on clustering solutions containing 2–8 dynamic states to provide a broad survey of the solution space. In the present data, these solutions captured significant aspects of dynamic variation in network connectivity. However, the focus on 2–8 brain states should not be taken to imply that meaningful properties are absent in alternative solutions.

### Brain states exhibit hierarchical features

**Fig. 2:**
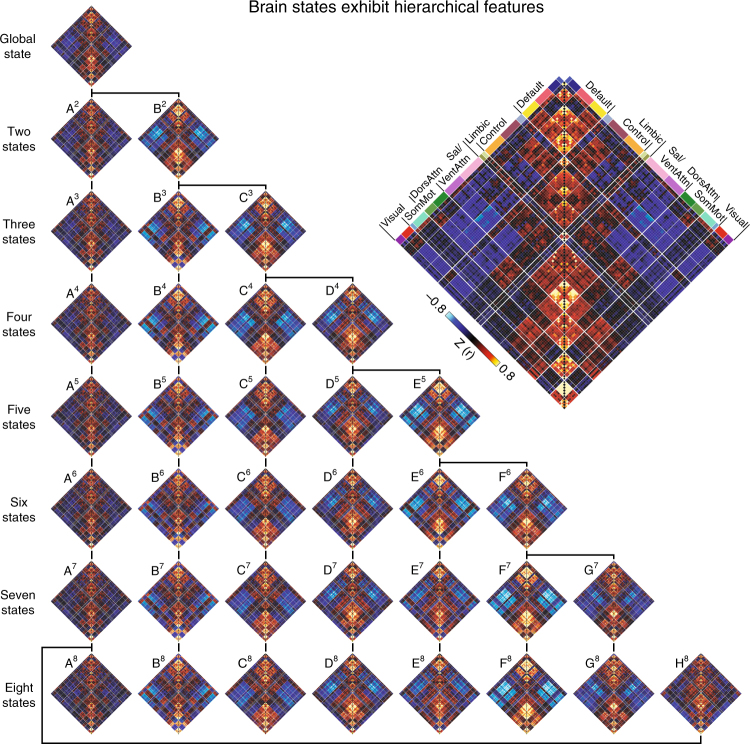
Brain states exhibit hierarchical features across distributed networks. Correlation matrices for state solutions 1 through 8 are shown for each region^39^. To quantify hierarchical relationships, an exhaustive search was performed to examine the scenario that two states of the (S + 1) state solution were subdivisions of a component of the S-state solution. Hungarian matching was used to determine which two-state combination in S + 1 was best matched to the S-state solution (see Supplementary Figure 4). Values reflect z-transformed Pearson correlations between every region and every other region. DorsAttn indicates dorsal attention, Sal salience, SomMot somatomoto, VentAttn ventral attention

Previous studies of functional network dynamics have largely focused on single clustering solutions in isolation, choosing a fixed number of states a priori. The relations between these isolated dynamic network configurations and other possible solutions remain unclear. To address this question, we employed a matching analysis examining the preservation or fractionation of individual brain states as the complexity of our clustering solution increased. An exhaustive search was performed exploring the scenario that two states in solution S + 1 were subdivisions of a state within solution S. Hungarian matching (Supplementary Figure 2) was used to determine which two-state combination in S + 1 was best matched to the S-state solution by minimizing network dissimilarity. As solution complexity increased, a hierarchical structure was observed, with some network configurations preserved across levels (Fig. 2; see Methods, Hierarchy analysis) and selected hybrid states in solution S breaking into substates within solution S + 1. A single state, termed state A, was evident across state solutions 2 through 7, not splitting to produce a hybrid substate until the solution complexity increased to eight states. These analyses demonstrate the stable expression of canonical network configurations across a variety of state solutions (2–8). Of note, when larger numbers of states were considered, increasing solution complexity was reflected in the hierarchical fractionation of particular states into substates.

Static analyses of network function suggest that heteromodal association cortices are more functionally variable than the unimodal cortex across the population^46^. These aspects of the cortex, and its associated networks, are implicated in a host of complex cognitive functions^47^ and overlap with regions that predict individual differences in behavioral performance^46^. We then explored if the heteromodal association cortex also exhibits heightened dynamic variability, as reflected in fluctuating connectivity configurations over time. Analyses examining the variance of mean network connectivity across the state solutions revealed evidence for dissimilar patterns of network expression across the dynamic states (ANOVA of coefficient of variance with state solutions treated as repeated measures, F_15_ = 44.11, *p* ≤ 0.001). Consistent with reports of heightened time-resolved network flexibility within the association cortex^13^, the greatest between-state variability was found in aspects of default, attention, and control networks (Supplementary Figure 3).

### Evidence for an attractor state

**Fig. 3:**
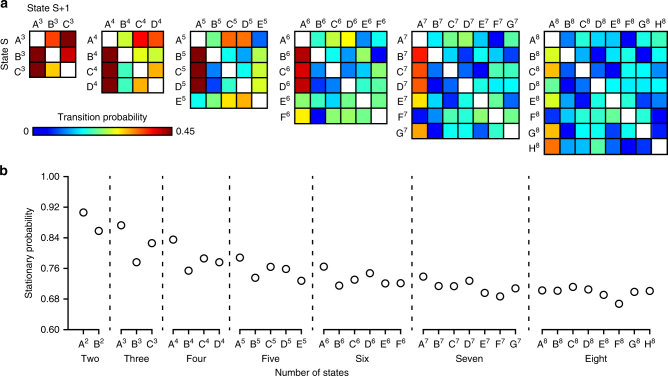
Evidence for an attractor state. **a** When transitioning between states, participants display an increased probability of entering state A. The probability of transitioning to each state is shown for all nonredundant possible combinations across state solutions 3–8 (1, 2 are obligated). Each row sums to 1. **b** State A displays the increased probability of remaining in the same state for solutions 2–5 (*p*s ≤ 0.001). The graph reflects the tendency for states in solutions 2–8 to show steady-state behavior, or the probability of remaining in a given state from time point *t* to time point *t*+1

In addition to characterizing the profiles of network connectivity across clustering solutions, brain dynamics can be studied in terms of the relative likelihood of transitions occurring among locally stable states over time^7,16,17^. To assess this feature of temporal organization, we examined the probability that participants would shift from a given state S to a different state (transition probability), as well as the probability that they would remain in state S. Window-by-window estimates were created for each state solution by matching the participant-specific connectivity matrices to the group atlas of brain states. This generated a vector of 110 expressed states for each participant. As solution complexity increased, participants were more likely to transition into (*p*s ≤ 0.001), and remain in state A (all test states 2–5 *p*s ≤ 0.001; states 6–8 *p*s ≥ 0.05; Fig. 3), termed the “attractor state.” Dwell time was calculated as the percent of the total time a given participant expressed state S relative to the total time in states not-S. Across the clustering solutions, the observed brain states exhibited quasi-stable expression, with all states maintaining nonzero dwell times (*p*s ≤ 0.001). Reflecting the transition probability analyses detailed above, dwell times were nonuniform across brain states. Participants dwelled most in attractor state A (*p*s ≤ 0.001) relative to all other states.

**Fig. 4:**
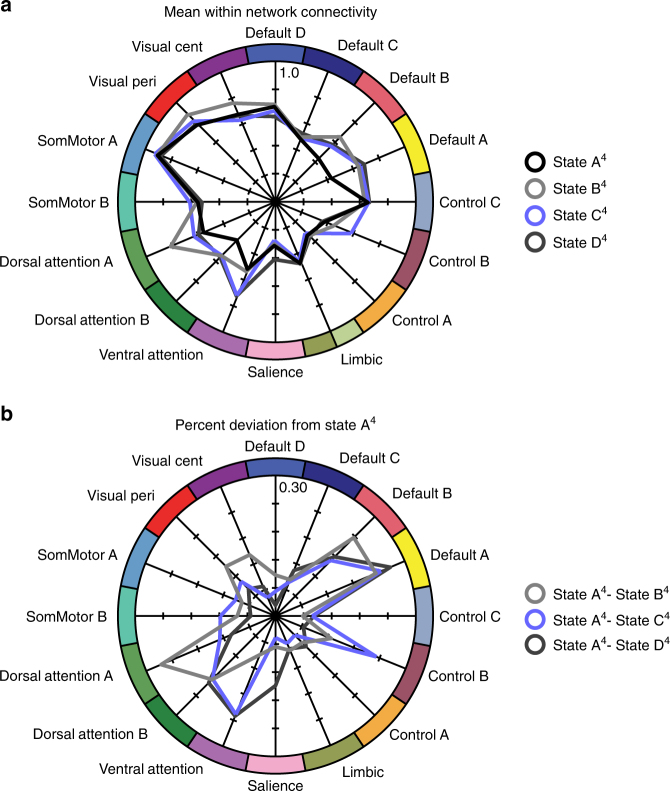
Profiles of within-network connectivity across the four-state solution. **a** The mean within-network connectivity is shown across each state of the four-state solution (*n* = 1919). Values reflect z-transformed Pearson correlations. **b** Percent deviation from state A^4^. Values reflect the percent change in mean network connectivity of states B^4^–D^4^, relative to state A^4^

Several core patterns that distinguish brain states across clustering solutions were evident. For ease of interpretability, the four-state solution was selected to characterize these features of dynamic connectivity (Fig. 4). As noted in the Hungarian matching analyses detailed above (Fig. 2), state A^4^ most closely resembled the global state revealed through traditional static analyses of network function. Across network configurations, state A was typified by a relatively flattened profile of connectivity (Fig. 4a). In line with this muted connectivity profile, the expression of state A became increasingly frequent in the second relative to the first half of the scan across each clustering solution (2–8 states; *t*s_1919_ ≥ −6.07, *p*s ≤ 0.001), potentially linking with shifts in arousal and vigilance^14,15^.

The remaining states varied markedly from the state A^4^ connectivity pattern with differences evident across states both within and between functional networks (Fig. 4b). Relative to state A^4^, states B^4^, C^4^, and D^4^ were characterized by increased expression of default A (see Fig. 1a for network topographies), with state B^4^ also marked by heightened within-network visual system and default B connectivity. State B^4^ notably differed from other network configurations in its recruitment of dorsal attention network A. Conversely, states C^4^ and D^4^ displayed increased correlations within the ventral attention network. There was also striking variation in the coupling of the frontoparietal network, with state C^4^ primarily characterized by increased connectivity in the control B network.

States B^4^, C^4^, and D^4^ showed marked departures from the off-diagonal coupling evident in state A^4^ and the global state (Fig. 2). The negative correlations between the default and somatomotor networks were attenuated in state B^4^ and were more evident in states C^4^ and D^4^. While states B^4^ and C^4^ displayed heightened correlations linking default and control networks, state D^4^ exhibited increased connectivity between default and the salience, attention, and somatomotor systems. Additionally, state C^4^, and to a lesser extent state D^4^, displayed increased cross-network connectivity between the attention, somatomotor, and visual networks. Together, these varying network configurations demonstrate nonrandom departures from connectivity patterns observed in the global state. There is a strong correspondence between the structure of intrinsic and extrinsic (task-evoked/coactivation) networks of the human brain, suggesting that the topological characteristics of the brain at rest are closely linked to cognitive function^12,48^. The transient expression of integrated network configurations may enable fast and accurate cognitive task performance^49^. Emerging evidence suggests that an individual’s unique profile of dynamic network connectivity could provide novel insights into the study of behavioral variability across both health and disease. In this regard, a crucial step is the characterization of intra-subject reliability and inter-subject variability of the observed profiles of time-varying brain organization across the population.

### Network dynamics are a marker of individual differences

**Fig. 5:**
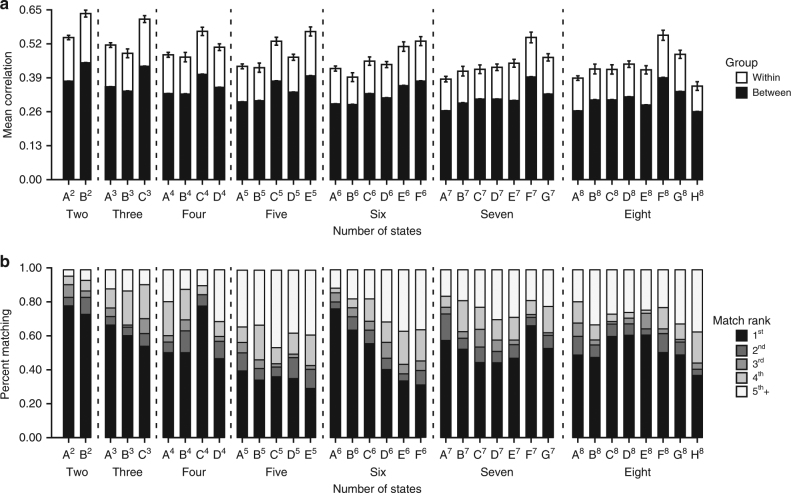
Brain states are reliably expressed and can be used to identify individual participants. **a** Mean rho values within and between subjects across visits for all stable dynamic states. Correlation matrices from participants who had at least two scans within 6 months of each other (*n* = 79) were concatenated, and a Pearson correlation was computed for scan 1 relative to scan 2. **b** Permutation and correlation analyses^27^ revealed that dynamic states are consistent within individuals over time. Identification rank accuracy is shown for each state in a subgroup of participants scanned on two different days within 6 months (*n* = 79). Rank denotes the order of similarity of a participant’s visit 1 to their visit 2, with 1 being a perfect identify match

Static descriptions of brain functional organization act as a biological fingerprint that can identify specific individuals within a larger group^27–29^. We aimed to determine whether the observed dynamic states were reliably expressed in a manner that would allow us to characterize unique within-subject profiles of transient network organization. To this end, we first assessed within-session reliability. For participants with two bold runs (*n* = 1341) in the same scanning session, correlation matrices were concatenated for each individual brain state, and a Pearson correlation was generated for run 1 relative to run 2. *T*-tests were used to compare the distributions of the resulting *r* values within and across individuals. For each state, we observed greater within-participant similarity for same-day scans (*r*s ≥ 0.49) compared to between participants (*r*s ≤ 0.23, 2–8 states; *p*s ≤ 0.001; see Methods, Individual identification analyses; Supplementary Figure 4). We then examined a cohort of participants with two scan visits collected on different days (≤6 months apart; mean = 63.35 ± 48.10 days; *n* = 79). Analyses demonstrated consistent within-subject dynamic state expression across visits (Fig. 5a; *p*s ≤ 0.001). Suggesting relatively stable intra-subject reliability across time, the observed within-participant similarity of the expressed brain states (Fig. 5a) did not vary as a function of the number of days between participant visits (absolute value of *r*s ≤ 0.21; *p*s ≥ 0.09).

Permutation tests^27^ were used to assess if dynamic connectivity profiles act as a signature that can accurately identify individual participants from a larger group. Correlation matrices from visit 1 and visit 2 were iteratively shuffled across participant labels and examined relative to their correct pairing. Identification was considered correct if the true visit 1 and 2 pair were maximally similar to each other. Correct identification was evident within participants relative to the shuffled list across all state solutions (*p*s ≤ 0.01; see Fig. 5b), suggesting that it is possible, with high accuracy, to identify an individual from a large group of participants solely on the basis of their dynamic connectivity profile. Dwell time was also more similar within, relative to between, participants for scans on the same day and across visits (all *p*s ≤ 0.01). These findings provide the first evidence to suggest that, like static characterizations of intrinsic connectivity, an individual’s dynamic brain function represents a unique and reliable biological signature, highlighting the potential use of intrinsic network dynamics as a neural marker of individual differences across both health and disease.

### Preferential state and network disruptions in psychosis

Impairments in the integration and processing of information across large-scale distributed brain networks are thought to mark psychotic disorders (including schizophrenia, schizoaffective disorder, and psychotic bipolar disorder)^30,50,51^. Converging evidence suggests that these disruptions may reflect alterations in the temporal dynamics of brain function^8,16,17,35^. We next examined the expression of time-varying network configurations in a cohort of patients with psychosis (*n* = 179) and a demographically and data-quality-matched comparison sample (*n* = 369). To evaluate the integrity of dynamic brain functions in these groups, we created windowed correlation matrices for each participant. For simplicity, we only consider the four-state solution, focusing on four states due to the stability of the associated clustering solution (Supplementary Figure 1) and the relatively high within-participant reliability (Fig. 5 and Supplementary Figure 4). Importantly, while we discuss the four-state solution, meaningful properties are likely present in other dynamic network configurations. We matched each windowed participant-specific matrix to the dynamic states established in our previous analyses and calculated mean correlation matrices across both groups. Following the correction for nuisance variables (motion, age, sex, handedness, and scanner bay), group differences were calculated for each of the four states for dwell times and network connectivity [false-discovery rate (FDR), *α* ≤ 0.05].

**Fig. 6:**
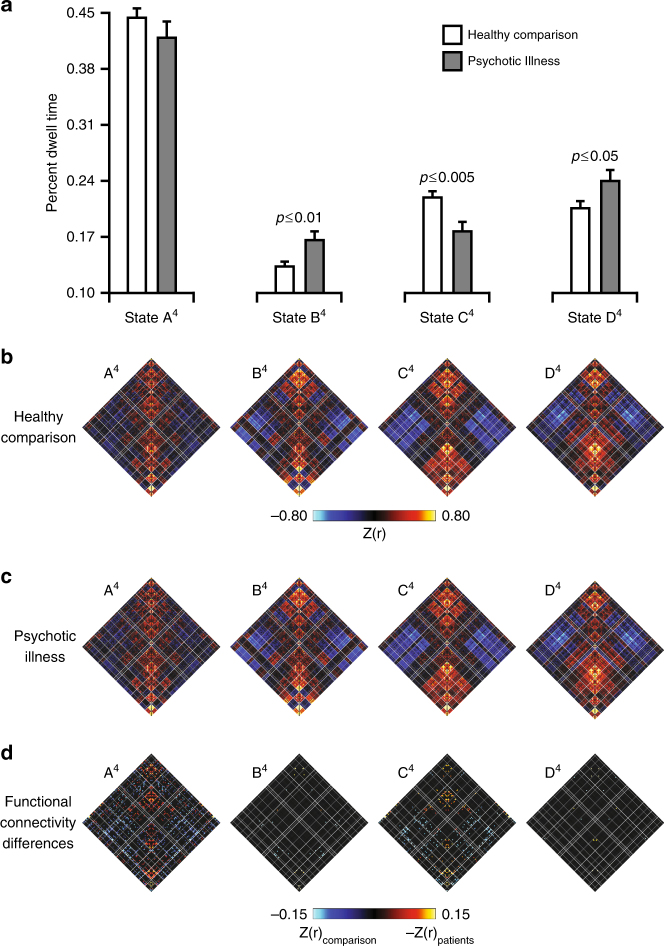
Psychotic illness links to state-specific reductions in connectivity. **a** Bar graphs display the percent of time healthy comparison participants and individuals with psychotic illness dwell in each state of the four-state solution. **b**–**c** The 2D grids (**b**–**c**) display the complete coupling architecture of the cerebral cortex measured at rest for each group of participants. **d** Differences were obtained by an analysis of variance of z-transformed Pearson correlation values after linear regression of the effects of age, sex, race, ethnicity, and handedness [r(z)_Comparison_ – r(Z)_Patient group_]. The bottom panel shows significant group differences at false-discovery rate *q* ≤ 0.05

Altered intrinsic connectivity in patients with psychosis observed in traditional static analyses may reflect both impaired functions within a specific brain state and the reduced tendency to enter particular network configurations over time. Analyses of dwell times did not reveal group variability in state A^4^ (*t*_537_ = 1.12, *p* = 0.26; Fig. 6a). However, patients displayed increased dwell time in state B^4^ (*t*_537_ = −2.8, *p* ≤ 0.005) and nominally significant increases in state D^4^ (*t*_537_ = −2.13, *p* ≤ 0.03). Notably, patient dwell times were reduced in state C^4^ (Fig. 5; *t*_537_ = 2.98, *p* ≤ 0.003), which is characterized by increased frontoparietal control network connectivity relative to other states (Fig. 4).

**Fig. 7:**
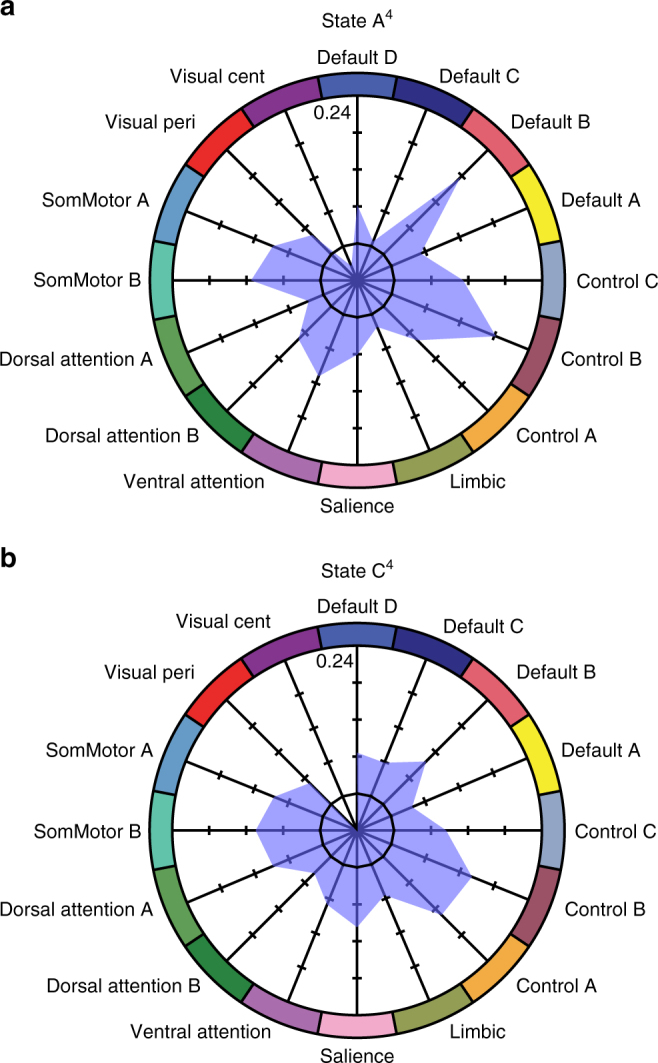
State-specific and state-general profiles of network dysregulation in psychosis. **a**, **b** Polar plots show percent difference in mean network connectivity between healthy comparison participants and individuals with psychotic illness for (**a**) state A^4^ and (**b**) state C^4^ (range: –6 to 24%). The black hexadecagon reflects the mean network correlations for the healthy comparison sample, set to zero. Values outside the hexadecagon reflect decreased correlation strength for patients, relative to the healthy comparison sample

Analyses of region-to-region correlation strength across the four-state solution yielded sparse group differences in states B^4^ and D^4^. Conversely, widespread group differences were observed in states A^4^ and C^4^ (Fig. 6). Analyses of state A^4^ revealed reduced within-network correlations across the cortex, including in default and frontoparietal networks. The largest-magnitude differences in state C^4^ were localized to the frontoparietal control network (Figs. 6 and 7). Replicating prior reports^18^, states A^4^ and C^4^ were associated with less negative, or muted, correlations linking the aspects of the association cortex to other networks in psychotic illness.

**Fig. 8:**
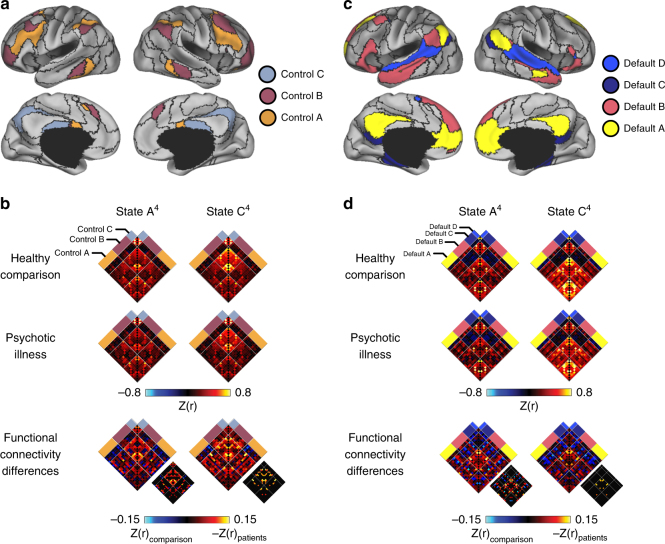
Psychosis is associated with reduced network connectivity in states A^4^ and C^4^. **a** The colored aspects of the cortex reflect regions estimated to be within the A, B, and C aspects of the frontoparietal control network^39^. **b** Functional connectivity matrices for the 50 left and right hemisphere regions of the frontoparietal control network shown for the healthy comparison and patient groups in states A^4^ and C^4^. Functional connectivity difference matrices were obtained by an analysis of variance of z-transformed Pearson correlation values after linear regression of the effects of age, sex, handedness, and scanner bay. Group differences significant at false-discovery rate *q* ≤ 0.05 are shown in each panel to the lower right of the unthresholded matrix. **c** The colored aspects of the cortex reflect regions estimated to be within the A, B, C, and D aspects of the default network. **d** Corresponding connectivity matrices are shown in 52 left and right hemisphere regions of the default regions for states A^4^ and C^4^. Reduced default network connectivity in psychosis is preferential to state A^4^

Follow-up analyses of state A^4^ and C^4^ revealed evidence for both state-general and state-preferential impairments in network connectivity (Fig. 7 and Supplementary Table 1). Within the frontoparietal control network, patients exhibited distributed deficits across states A^4^ (state A^4^ control B and C: *t*s_535_ ≥ 4.12, *p*s ≤ 0.001) and C^4^ (state C^4^ control B: *t*_513_ = 4.85, *p* ≤ 0.001; Fig. 8a–b). All other subnetworks failed to pass multiple-comparison correction (Bonferroni *p* ≤ 0.05; *p*s ≥ 0.01). Conversely, reduced default network connectivity in psychosis was observed in default B in state A^4^ (state A^4^ default B: *t*s_535_ = 6.08, *p* ≤ 0.001; default A, C, and D: *t*s_535_ ≤ 1.69, *p*s ≥ 0.09; Fig. 8c–d). No group differences in the default network survived correction for multiple comparison when considering state C^4^ (state C^4^ default B: *t*s_535_ = 2.91, *p* ≥ 0.01; default A, C, and D: *t*s_535_ ≤ 1.31, *p*s ≥ 0.19). These analyses suggest that prior observations of default and control network disruptions in psychosis may reflect temporally specific impairments, preferentially manifesting during the expression of transient configurations that recruit the function of these networks.

### Select network configurations mark active psychotic symptoms

Together, the analyses above suggest that time-varying network configurations capture stable aspects of inter-subject variability (fingerprints), while also serving to mark the presence of psychiatric illness. These findings suggest that dynamic approaches may provide critical information that can help predict the presence of distinct clinical profiles within individuals, revealing symptom-relevant features of the disease^16,17^. To determine whether individual differences in time-varying profiles of connectivity are relevant to clinical symptomatology, we investigated the extent to which specific brain states can identify patients who experienced active psychotic symptoms at the point of initial assessment. To this end, we examined clinician reports of the current psychotic symptoms in our patient sample, assessed through the DSM-IV (SCID) clinician-rated presence of delusions and/or hallucinations in the past month^38^, and selected participants who expressed all four brain states (*n* = 130). Using a machine-learning-based framework, elastic net logistic regression, we demonstrated that the presence of active psychotic symptoms can be predicted based solely on the dynamic connectivity profile of previously unseen individuals (Methods, Prediction of active psychotic symptoms)^52,53^. First, training a model in 91 participants (70% of the available patient sample), we used the edge strength from the dynamic network configurations of states A^4^, B^4^, C^4^, and D^4^ as candidate features upon which we conducted variable selection. The extracted features were then used to predict the presence of active psychotic symptoms in individual participants, using leave-one-subject-out cross-validation. The fitted model was prospectively applied to edge strength of the dynamic network configuration from a held-out set of participants, and yielded a scalar probability measure for each individual, which constituted the predicted likelihood of active psychotic symptoms.

**Fig. 9:**
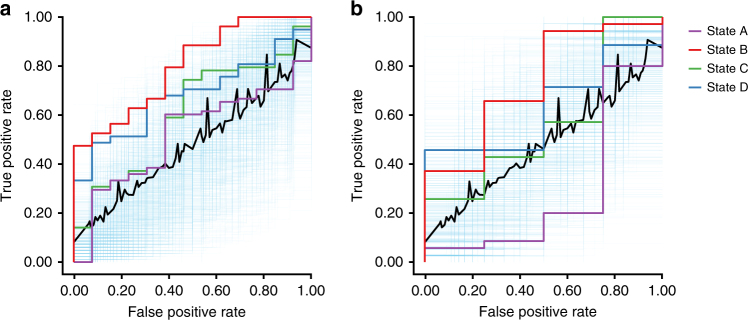
Specific brain states are predictive of distinct clinical symptoms in novel individuals. **a** State B^4^ uniquely predicted the presence of active psychosis in patients with psychotic illness. Results from a leave-one-subject-out cross-validation elastic net logistic regression analysis comparing predicted and observed psychotic symptoms (*n* = 91). The displayed area under the receiver-operating characteristic curve (AUC) reflects the scalar probability measure for each individual, predicting the likelihood of active psychotic symptoms. The blue semitransparent lines reflect 1000 ROC curves from 1000 permutation experiments. The black dashed line indicates the mean of these 1000 curves, approximating a null curve. The purple, red, green, and orange curves correspond to states A^4^, B^4^, C^4^, and D^4^, respectively. **b** Connectivity models defined on training data predict the presence of active psychotic symptoms in an independent group of participants (*n* = 31). The displayed AUC graph reflects the scalar probability measure, predicting the likelihood of active psychosis, defined using positive edges from the initial training set without further fitting or modification, in a held-out sample of patients

Consistent with an increased tendency to enter state B^4^ in patients, relative to other network configurations (Fig. 6a), state B^4^ specifically predicted the presence of active psychotic symptoms in the training set (positive network predictive network: AUC = 0.80, *p* ≤ 0.001, permutations = 5000; Fig. 9a). As a comparison, the AUC values using a positive network of states A^4^, C^4^, and D^4^ were 0.60 (*p* = 0.16), 0.68 (*p* = 0.05), and 0.52 (*p* = 0.37), respectively. A similar, although subtler, predictive profile was observed for the negative networks in the training set where state B^4^ predicted the presence of active psychotic symptoms (AUC = 0.724, *p* ≤ 0.05). The remaining negative network AUC values for states A^4^, C^4^, and D^4^ were 0.39 (*p* = 0.74), 0.50 (*p* = 0.43), and 0.56 (*p* = 0.26), respectively.

Next, we demonstrated that predictive symptom models derived from dynamic network configurations can generalize to data from novel individuals, applying the predictive network models in the left-out patient sample (30% of the available patient sample). Here, without further fitting or modification of the initial models, we tested the neurological signatures identified in the training set for the predication of active psychotic symptoms in a held-out group previously unseen subjects (*n* = 39). The negative-network models did not generalize to the held-out sample (AUCs < 0.66, *p*s > 0.13). The previously identified positive-network model for state B^4^ served as a generalizable predictor of active psychotic symptoms in an independent sample (AUC = 0.74, p ≤ 0.05, permutation *n* = 5000; Fig. 9b). As a comparison, the AUC values derived across dynamic states A^4^, C^4^, and D^4^ for out-sample predication were 0.56 (*p* = 0.32), 0.63 (*p* = 0.18), and 0.29 (*p* = 0.92). Highlighting that dynamic analyses may reveal information hidden in traditional static analyses of network function, models derived from the static or global state were not predictive of active psychosis in either the training (positive network: AUC = 0.62, *p* = 0.12; negative network: AUC = 0.64, *p* = 0.09) or test samples (positive network: AUC = 0.49, *p* = 0.53; negative network: AUC = 0.24, *p* = 0.96).

Patients expressing active psychotic symptoms, relative to those without, presented with increased positive (present: 19.90 ± 5.67, absent: 8.88 ± 2.20; *t*_128_ = 7.91, *p* ≤ 0.001), negative (present: 13.98 ± 7.76, absent: 10.18 ± 3.93; *t*_128_ = 1.98, *p* ≤ 0.05), and general psychopathology (present: 31.46 ± 7.81, absent: 26.53 ± 7.74; *t*_128_ = 2.43, *p* ≤ 0.05) symptoms (Supplemental Fig. 7) as assessed through the positive and negative syndrome scale (PANSS)^54^. To establish the specificity of the predictive model for the presence of psychotic features, we examined PANSS scale scores in the left-out patient sample. The previously identified positive-network model for active psychosis in state B^4^ served as a generalizable predictor of PANSS positive-scale symptom severity in the independent sample (AUC = 0.72, *p* ≤ 0.05). The observed effect was preferential to the state B^4^ network model. The associated AUC values derived across dynamic states A^4^, C^4^, and D^4^ for positive out-sample predication were 0.51 (*p* = 0.46), 0.66 (*p* = 0.10), and 0.33 (*p* = 0.90). As above, the negative-network models did not generalize to the PANSS positive-scale scores in the held-out sample (AUCs < 0.67, *p*s > 0.09). Suggesting a degree of symptom specificity, the PANSS negative (positive network: AUC = 0.41, *p* = 0.83; negative network: AUC = 0.52, *p* = 0.42) and general psychopathology scale (positive network: AUC = 0.62, *p* = 0.21; negative network: AUC = 0.41, *p* = 0.74) scores were not predicted by the state B^4^ model, or the models resulting from the other states (positive network: AUCs < 0.51, *p*s > 0.48; negative network: AUCs < 0.59, *p*s > 0.18). These results provide preliminary evidence that the presence of clinical symptoms, in this case, active psychosis, may be associated with predictable patterns within an individual’s time-varying network profile, suggesting that dynamic approaches have potential utility for predicting a wide range of clinical symptoms and cognitive abilities.

## Discussion

The functional coupling of cortical regions varies in response to explicit task demands^5^ and in conjunction with shifts in arousal^13^, attention^14,15^, and markers of autonomic activity^24^. Here, using a sliding-window approach on resting-state imaging data, we demonstrated that cortical brain networks possess a time-varying organizational structure with hierarchical properties. Select network configurations fractionated into substates in an ordered manner, and a global attractor state (termed state A) was evident across clustering solutions (2–8 states). Suggesting that profiles of dynamic network connectivity may link to behavioral differences in health and disease, the observed brain states reflected individually specific signatures, or fingerprints, of time-resolved connectivity that were unique and reliable within participants across both scans and independent visits separated by up to 6 months. Extending upon prior static analyses of network function^27–29^, we found that it is possible, with high accuracy, to identify specific individuals from a large group of participants solely on the basis of their profiles of dynamic connectivity. Patients with schizophrenia and psychotic bipolar disorder exhibited state-specific, intermittent disruptions within cortical association networks believed to mark the presence of psychotic illness. Finally, our analyses revealed that individual variability in select network configurations (state B^4^) can be used to predict the presence of active psychotic symptoms in novel participants. Together, these results highlight the potential to discover individualized dynamic network profiles that are predictive of cognitive abilities and clinical symptoms across health and disease.

Spontaneous brain activity is constrained, but not fully determined, by structural connectivity^55–59^. This raises the possibility that a quasi-stable functional architecture may anchor on anatomic connectivity, with transient network configurations reflecting the influence of momentary cognitive processes, environmental demands, or other biological information. Supporting this conjecture, while functional coupling occurs in the absence of cognition^41^, under general anesthesia, brain activity exhibits a reduction in spontaneous transitions across network configurations^60^, settling into a restricted dynamical repertoire that closely resembles a fixed network defined by structural connectivity^56^. The present analyses indicate the expression of core, or canonical, time-varying network configurations that separate in an ordered manner across increasingly complex clustering solutions. Throughout the hierarchy, a multistable dynamical system was evident, fluctuating around a global attractor state (state A) that possesses a relatively muted connectivity profile. Decreased within-network coupling is evident when spontaneous neuronal activity adheres to fixed correlation configurations defined by structural connectivity^61,62^. Although speculative, our results suggest the presence of an attractor state that could preferentially link to the large-scale anatomical structure of the human cerebral cortex. Future cross-modal analyses will be necessary to explore these hypotheses and test the extent to which patterns of spontaneous brain activity might reconfigure around an underlying anatomical skeleton.

Regions within the association cortex display increased functional flexibility, potentially serving to integrate information across more specialized aspects of the cortex^63^. This profile of malleability is reflected in static analyses of intrinsic network function where heightened population-level variability has been observed in association relative to unimodal cortices^46^. Consistent with prior reports of marked heterogeneity in the dynamic flexibility of neural regions^13,64^, we observed the greatest cross-state variability within aspects of default, attention, and control networks. The trade-off between control and attention systems is thought to be a central feature of many cognitive functions, including adaptive goal pursuit, working memory, and set shifting^65^. While we are unable to make direct claims regarding the association between time-varying profiles of network function and cognition, converging evidence suggests that broad properties of network connectivity may be preserved across experimental contexts (e.g., intrinsic or task-evoked)^12,48^ and analysis strategies^48^. An important area of future work will be to establish the extent to which the cortical network structure adjusts as a function of task and/or environment. One speculative possibility is that transient periods of strong within-network coupling may correspond to epochs of high efficiency, a property of brain organization theorized to optimize communication across distinct functional domains^66^.

Human cognition is a fluctuating process, and there is growing evidence that functional connectivity patterns exhibit complex spatiotemporal dynamics at multiple time scales^58^. Time-varying brain states, such as those identified in the present analyses, have been hypothesized to reflect changes in the ongoing cognitive processes during rest^7^. However, the existence, putative origins, and cognitive correlates of dynamics in resting-state fMRI remain a topic of empirical debate^10,11,19,67^. While the relations linking intrinsic time-varying network profiles with cognition remain speculative, there are two commonly held views. First, that by applying clustering algorithms, the brain’s dynamic architecture can be carved at the joints, revealing discrete brain states. Second, that the resulting macro-level brain states index, or enable, distinct biological and cognitive processes. In the current analyses, we apply k-means clustering to identify dissociable network configurations in solutions from 2 to 8 brain states. Critically, we do not claim that these configurations reflect wholly distinct brain states separated by sharp transitions, and we are not in the position to ascribe associated cognitive functions. Rather, the data are consistent with the possibility that large-scale network coupling gradually fluctuates around a core functional architecture. However, the extent to which neurobiological and cognitive mechanisms may drive the observed transient connectivity patterns remains an open question.

Debates regarding the existence of discrete brain states should not be taken to imply that temporal fluctuations in network connectivity lack biological information. Notably, the present analyses indicate that an individual’s profile of dynamic functional connectivity is unique and stable over the course of days and months. Patterns of connectivity defined through traditional static analyses are heritable^25,26^ and function as a trait-like signature that can accurately identify participants from a large group^27–29^. While prior demonstrations of cross-session identification were established when participant visits were separated by a single day^27^, these data suggest that single measures of time-averaged connectivity may provide meaningful trait-like information about individuals. Here, we observed robust participant-specific matching when visits were separated by up to 6 months (mean = 63.35 ± 48.10 days apart, range = 2–151 days). Despite some variability, identification accuracy was not limited to any one state or clustering solution, indicating that participants may be identified with relatively thin slices of transient brain activity. These discoveries highlight the potential to identify associations linking the unique dynamic functional architecture of an individual’s brain to the integrity of large-scale corticocortical pathways^29^. The continued development of time-varying data analytic approaches with high sensitivity to individual variability could facilitate the discovery of meaningful biomarkers for both cognitive ability and disease states.

Schizophrenia and psychotic bipolar disorder are marked by altered intrinsic network connectivity, potentially contributing to widespread changes in information processing^30–34^. A key question facing the field is the extent to which the temporal organization of the brain is impaired in psychotic illness^8,16^. The present analyses reveal the presence of both state and network preferential reductions in functional connectivity. Patients with schizophrenia spend more time than healthy individuals in network configurations typified by reduced large-scale connectivity, while also showing muted negative correlations between default and other networks^18^. In line with this literature, we observed a general profile of decreased within- and increased between-network correlations. Our analyses were also consistent with prior work, demonstrating executive functioning and cognitive control abnormalities in patients with psychotic illness^68^, revealing preferential disruptions in frontoparietal control network connectivity in the attractor state (state A^4^) and state C^4^, a network configuration marked by increased within-network frontoparietal connectivity in healthy young adults. This profile of fluctuating abnormalities in network connectivity was also evident in a default network, which exhibited state-preferential impairments in state A^4^. Critically, these analyses do not demonstrate selective abnormalities in discrete network configurations that are specific to psychotic illness. Rather, consistent with evidence for alterations in dynamic brain architecture in patient populations^16–18^, they suggest that broad disruptions across cortical association networks in psychosis may emerge through transient abnormalities preferentially evident during the expression of particular network configurations^17,35^. Together with growing evidence linking behavior to temporally derived network configurations^12–15^, the presence of both individual specificity (fingerprints) in brain dynamics across the population and evidence for fluctuating, network-specific impairments in patients with severe psychopathology have strong implications for clinical practice. For instance, these data suggest a potential avenue to identify distinct symptom profiles, track time-varying disease states, responses to environmental perturbations, or individually specific treatment responses.

Consistent with the aim of delineating disease-relevant markers of brain biology, the current analyses suggest that models based on transient profiles of network function may serve as powerful, generalizable predictors of clinical symptomatology. In a group of patients with psychotic illness, we identified a specific time-varying network profile whose strength predicted the presence of active psychotic symptoms at the point of clinical assessment. This whole-brain network model provides preliminary evidence for meaningful, clinically relevant signals in patterns of dynamic intrinsic connectivity. Suggesting that the clinically relevant features of intrinsic brain dynamics are robust and generalizable, networks defined within an initial training set successfully predicted active psychosis in a completely independent sample. As reflected in our analyses, a randomly selected, previously unseen, patient with active psychosis could be distinguished from another patient without psychosis at ~74.0% accuracy based on their dynamic expression of selected network configurations (state B^4^), demonstrating a relatively high level of precision. Critically, these relations were not evident through traditional static analysis. Readers should note that these analyses leverage cross-sectional data, and are technically postdictive. Additional research should assess if this cross-sectional/retrospective approach generalizes to the prospective prediction of symptoms, prior to clinical assessment.

Suggesting a degree of specificity for the prediction of psychotic symptom severity, the active psychosis model served as a unique predictor of PANSS positive-scale scores (binarized as greater or less than one standard deviation below the sample mean) in the left-out patient sample, with poor prediction observed for both negative and general psychopathology symptoms. Caution is warranted given the limited sample size; however, these data provide evidence to suggest that dynamic network models trained on different, yet associated, symptoms have the potential to generalize across clinical measures. These data expand our current knowledge regarding abnormal large-scale network function in patients with schizophrenia and psychotic bipolar disorder, and highlight the use of dynamic analytic approaches when examining intrinsic connectivity across heath and disease. Taken together, the present analyses provide a preliminary proof of concept to suggest that the altered connectivity of specific transient network configurations may link to the expression of discrete symptom profiles. Future work should focus on the identification of relations linking functional network dynamics to the expression of psychological and behavioral aspects of illness.

In conclusion, we demonstrated the presence of a fluctuating and reconfigurable hierarchy across the functional connectome. The observed dynamic network profiles were unique and reliable within individuals over the course of months and impaired in patients with psychotic illness. Our analyses suggest that temporal patterns of connectivity between cortical regions link to the broad functional capacities of individual human brains, enabling the prediction of specific symptom profiles within patient populations. These data have important implications for the study of behaviors and features of psychiatric illnesses that possess time-varying patterns of expression.

## Methods

### Data acquisition

Native English-speaking young adults (aged 18–35) with normal or corrected-to-normal vision were recruited from Harvard University, Massachusetts General Hospital, and the surrounding Boston communities through an ongoing large-scale study of brain imaging and genetics (*n* = 1919; age: 21.35 ± 3.20; female: 56.53%; right handed: 92.40%)^43^. History of psychiatric illness and medication usage was assessed through a structured phone screen. On the day of MRI data collection, participants completed additional questionnaires concerning their physical health, past and present history of psychiatric illness, and medication usage. Exclusion criteria included a history of head trauma, current or past Axis I pathology, neurological disorders, current or past psychotropic medication use, current physical illness, and current or past loss of consciousness. Participants provided written informed consent in accordance with guidelines set by the Partners Health Care Institutional Review Board and the Harvard University Committee on the Use of Human Subjects in Research. For the present study, we assessed the extent to which the dynamic network architecture of the cortex is reliable within and across visits. To accomplish this, an additional data set (*n* = 79; age at the first scan: 20.99 ± 2.93; female: 45.56%; right handed: 89.87%) was acquired over the course of the primary collection effort. Data were collected on 2 independent days (mean = 63.35 ± 48.10 days apart; min = 2; max = 151).

Patients with psychotic illness were recruited from clinical services at McLean Hospital (*n* = 170; age: 32.08 ± 11.64; female: 66.47%; right handed: 84.71%), including 41 patients diagnosed with schizoaffective disorder, 56 with schizophrenia, and 73 with psychotic bipolar disorder. Study procedures are detailed in Baker et al. 2014^30^. Briefly, exclusion criteria included neurological illness, positive pregnancy test, electroconvulsive therapy in the last 3 months, and history of head trauma. Reflecting the severity of the present sample, the majority of patients (82%) reported experiencing active psychotic symptoms at the time of their clinical assessment, as assessed through DSM-IV (SCID) clinician-rated symptomatic diagnostic criteria, indicating the presence of delusions and/or hallucinations in the past month^38^. All patients were assessed for active symptoms within 24 h of scan using the Positive and Negative Syndrome Scale (PANSS; positive scale: 18.46 ± 6.51; negative scale: 13.48 ± 7.47; general psychopathology scale: 30.82 ± 7.95)^54^. A demographically matched healthy comparison sample was recruited from the surrounding Boston communities (*n* = 369; age: 37.16 ± 14.65; female: 62.06%; right handed: 91.33%). The McLean Hospital Institutional Review Board approved the study, and all participants provided written informed consent. The control group was significantly older than the patient group (t = –3.98, *p* ≤ 0.001). No significant group differences were identified in sex, handedness, or education (*p*s ≥ 0.07). The comparison sample was explicitly selected to match on the basis data quality. The BOLD runs for the patient and comparison groups did not differ in terms of slice-based temporal signal-to-noise ratio (comparison: 161.17 ± 46.05; patient: 158.35 ± 71.53) or the number of relative translations in 3D space ≥0.1 mm (comparison: 31.05 ± 29.15; patient: 32.29 ± 29.66; *p*s ≥ 0.58). The slice-based signal-to-noise ratio was calculated as the weighted mean of each slice’s mean intensity over time (weighted by the size of the slice).

All imaging data were collected on 3-T Tim Trio scanners (Siemens) with a 12-channel phased-array head coil at Harvard University, Massachusetts General Hospital, or McLean Hospital. Structural data included a high-resolution multi-echo T1-weighted magnetization-prepared gradient-echo image (TR = 2200 ms, TI = 1100 ms, TE = 1.54 ms for image 1–7.01 ms for image 4, FA = 7°, 1.2 × 1.2 × 1.2 mm, and FOV = 230). Functional data were acquired using a gradient-echo echoplanar imaging sequence sensitive to blood oxygenation level-dependent contrast with the following parameters: 124 time points; repetition time = 3000 ms; echo time = 30 ms; flip angle = 85°; 3 × 3 × 3-mm voxels; FOV = 216; and 47 axial sections collected with interleaved acquisition and no gap. Participants were instructed to remain still, stay awake, and keep their eyes open. Although no fixation image was used, participants with psychotic illness were monitored via eye-tracking video to ensure compliance during functional scans. One to two runs were acquired for each participant (70.89% of the main sample, 69.41% of patient participants, and 46.88% of the matched comparison received a second run). Software upgrades (VB13, VB15, and VB17) occurred during data collection. Reported results are after partialing out variance associated with scanner and software upgrade.

### Data preprocessing

Data were processed with a series of steps common to intrinsic connectivity analyses^69–71^. Preprocessing included discarding the first four volumes of each run to allow for T1-equilibration effects, compensating for slice acquisition-dependent time shifts per volume, and correcting for head motion using rigid body translation and rotation. Additional steps included the removal of constant offset and linear trends over each run, and the application of a temporal filter to retain frequencies below 0.08 Hz. Sources of spurious variance, along with their temporal derivatives, were removed through linear regression. These included six parameters obtained by correction for rigid body head motion, the signal averaged over the whole brain, the signal averaged over the ventricles, and the signal averaged over the deep cerebral white matter. Structural data and functional data were aligned as described in Yeo et al.^39^ and Buckner et al.^72^ using the FreeSurfer software package. This method yields a surface mesh representation of each participant’s cortex, which is then registered to a common spherical coordinate system. Images were aligned with boundary-based registration^73^ from the FsFast software. Functional and structural images were then aligned to the common coordinate system by sampling from the middle of the cortical ribbon in a single interpolation step to reduce blurring of the functional signal across sulci and gyri. A 6-mm smoothing kernel was applied to the functional data in the surface space, and data were downsampled to a 4-mm mesh. Additional details on the preprocessing procedures are detailed in Holmes et al.^43^ and Yeo et al.^39^.

### Dynamic connectivity sliding-window analysis

Cortical functional coupling matrices were computed for each participant, across all available parcels within the 17-network functional atlas of Yeo et al.^39^. We defined 114 regions (57 per hemisphere) that surveyed all 17 networks. Correlation matrices were constructed to include all region pairs arranged by network membership.

Connectivity across time was analyzed using a sliding-window approach (width = 33 s)^7,8^. Prior work suggests that a sliding-window range of 30–60 s is appropriate for dynamic connectivity analyses^74^. Pilot analyses (available upon request) revealed consistent state solution stability across varying sliding-window sizes of 33–63 s. Thirty-three-second windows were chosen in order to maximize signal estimates, while still capturing properties of transient functional connectivity^8,40^. A time series for each participant was extracted for the 57 regions in each hemisphere. Time-course correlations across 110 windows per bold run for each participant (220 windows if the participant had two runs) were calculated for each 114 × 114 region pair. To limit the redundancy across matrices and to reduce computational load, clustering was applied to a subsample of available windowed covariance matrices (1/10 windows). Results were consistent with the alternate approach of subsampling along the temporal dimension to identify windowed covariance matrices with local maxima in functional connectivity variance. The resulting correlation matrices were then aggregated and z-transformed prior to running the clustering analyses. The clustering analysis was iteratively applied to define distinct state solutions for 2 through 20 brain states. Additional details on the selected clustering approach are provided in Yeo et al.

As we did not have a priori hypotheses regarding the number of functional connectivity states, we assessed the stability of clustering solutions for all states 2–20. To do so, we examined the stability of the clustering analyses by iteratively and randomly splitting our data on two dimensions (sliding windows and pairwise connectivity) and rerunning the clustering solution 30 times^39^. The results were then compared using a Hungarian matching algorithm^75,76^, as described in the next section. Greater instability was quantified as a greater summation of deviation between the two cluster solutions (Supplementary Figure 1).

### Hierarchy analysis

To examine the relations linking each state in solution S with the states in solution S + 1, we used a Hungarian matching technique^75,76^. Every possible combination of states in state solution S + 1 was compared to each state in solution S. To illustrate this point, take the comparison of states in the three- and four-state solutions. Each possible combination in the four-state solution is established (A^4^B^4^, A^4^C^4^, A^4^D^4^, B^4^C^4^, B^4^D^4^, and C^4^D^4^) by calculating the mean of each cell in the 114 × 114 × n (here, *n* = 4) connectivity matrix across the combined states to create hybrid states. Next, each hybrid state is grouped along with the other states in the four-state solution and compared to the three-state solution (A^4^ + B^4^/C^4^/D^4^, then A^4^ + C^4^/B^4^/D^4^, then A^4^ + D^4^/B^4^/C^4^, etc.). Hungarian matching is used to determine which of the hybrid combinations in the four-state solution most closely approximates each state in the three-state solution (so, in the first example, the hybrid four-state solution comprised of A^4^ + B^4^/C^4^/D^4^ is matched to states A^3^, B^3^, and C^3^ in the three-state solution). This comparison is repeated until the match with the minimal cost is identified (Supplementary Figure 2).

### Identifying variability across defined state solutions

To determine the extent of network variability across brain states, we examined the variance of mean network connectivity in state solutions 2–8 (see Supplementary Figure 3 for variance with the four-state solution). We used ANOVA to assess between-state variability (coefficient of variance) with state solutions treated as repeated measures. The results confirmed that variance differed across the networks (F_15_ = 44.11, *p* ≤ 0.001). Post hoc tests revealed increased cross-state variability within default A and B relative to the default D, control A and C, limbic, somatomotor A, and visual B networks (Bonferroni-corrected *p*s ≤ 0.05, all other *p*s ≥ 0.5). Control B demonstrated greater variance relative to default C and D, control A and C, limbic, somatomotor, and visual networks (*p*s ≤ 0.05; all other *p*s ≥ 0.5). The salience/ventral attention and dorsal attention A networks exhibited increased variance relative to default C and D, control A and C, limbic, somatomotor, and visual networks (*p*s ≤ 0.05, all other *p*s ≥ 0.5).

### Individual identification analyses

To examine the extent to which the observed dynamic connectivity profiles are reliably expressed across scans and visits, we first selected participants with two bold runs (*n* = 1361). Next, we examined the 79 participants, set aside from the original cohort, who had two separate study visits within 6 months of each other. To test the relations within each individual’s profile of network dynamics for the two bold runs within the same visit, and then for the individuals with more than one visit, we implemented the following analysis: First, we obtained each participant’s state expression across time. To accomplish this, we used a Hungarian matching algorithm, assigning each time point in a participant’s windowed time-course data to individual states in the desired population-level state solution. For instance, in the four-state solution, we found the best fit for each window in the participant’s data and classified it as state A^4^, B^4^, C^4^, or D^4^. This yielded a vector of states for each participant, representing the participant’s state expression over the course of the scan. Following this, we took the average correlation matrix for each window within a state. So, for participant 1, we collapsed across all of the state A^4^ windows, and generated a mean for state A^4^, creating a 114 × 114 × S average state matrix for each participant. Participant matrices were vectorized, and Pearson correlations were run across every participant in two analyses for (1) bold 1 and bold 2; and every participant for (2) visit 1 and visit 2. Analyses indicated a significant level of consistency in within-individual connectivity profiles across bold runs and visits (Supplementary Figure 4). For each state solution, we ran a *t*-test for within-participant rho values and between-participant rho values. All tests revealed increased rho values within, rather than between, participants (all *p*s ≤ 0.001).

Permutation tests were performed in a manner consistent with prior studies examining functional connectome fingerprinting^27^. For these analyses, we considered participants with multiple study visits (*n* = 79) and utilized the state- and participant-specific connectivity vectors described above. A Pearson correlation was calculated across an ordered list for every participant’s connectivity vector from visit 1 compared to every other participant’s vector from visit 2. We first established a comparison distribution from chance. For each state, the participant vector from visit 1 was compared to a randomly permuted list of participants from visit 2, a Pearson correlation was calculated, a matching rank was assigned, and the identification rate was calculated. A “correct” identification was defined in cases where the highest-ranked rho value was within a participant (visit 1–visit 2) relative to other participants (for ranking results, see Fig. 4, main text). This was repeated 1000 times, and a *t*-test was performed comparing the number of identifications of the actual order participants relative to the distribution of the number of identifications in the permuted order of the participant list.

### Noise constraints

To assess the extent to which data quality might influence on our findings, we conducted a series of analyses aimed at detecting the differences in motion across state solutions. To obtain motion estimates by a participant for each window, we extracted the mean of the root mean square of relative motion for each participant across each window. We used the Hungarian matched state vector to classify relative motion within each TR to a state-specific window. We averaged motion values within states for each state solution. Although the relations linking state expression and motion were limited in size (*η*_p_^2^s ≤ 0.006), state A associated with the most motion for state solutions 2, 3, and 4 (all *p*s ≤ 0.05; Supplementary Figure 5). State A showed a muted increase in motion relative to other states in solutions 5 through 7 (five-state solution: *p*s ≤ 0.001 for state A^5^ relative to C^5^ and E^5^, *p* ≤ 0.05; *p* ≥ 0.59 for B^5^ and D^5^; six-state solution: *p*s ≤ 0.01 for state A^6^ relative to E^6^ and F^6^, *p*s ≥ 0.38 for B^6^, C^6^, and D^6^; seven-state solution: *p*s ≤ 0.001 for state A^7^ relative to E^7^ and F^7^; *p* = 0.07 for C^7^, *p* = 0.06 for G^7^, and *p* ≥ 0.61 for B^7^ and D^7^). In state solution 8, we found that state H^8^, a state that our hierarchy analyses identified as architecturally related to state A in solutions 2–7, was associated with more motion than all other states (*p*s ≤ 0.001).

Next, we examined the consistency of state stability in participants falling within the first and fourth quartiles of the distribution for motion (low motion: *n* = 424; high motion: *n* = 398). No group differences were identified in age, sex, or handedness (*p*s ≥ 0.59). To determine the stability of our state-clustering approach, we applied iterative k-means clustering to each group for each population-level stable state solution (2, 4, 5, and 8 states). To estimate the viability of the resulting solutions, data were resampled across sliding windows as described previously (Fig. 1, main text). A *t*-test was performed to assess the differences in the consistency sampling from the high and low motion groups. No differences in consistency were observed across groups (Supplementary Figure 6; all *p*s ≥ 0.43).

### Prediction of active psychotic symptoms

Here, we demonstrate that the strength of functional brain networks within specific brain states predicts the presence of active psychosis in previously unseen individuals. Elastic net logistic regression analyses were conducted with custom R code (*elasticnet* by Hui Zou, Trevor Hastie, and Robert Tibshirani). Elastic net regularization is a cross-validated regularized log-linear regression procedure that combines LASSO (least absolute shrinkage and selection operator) regularization and Tikhonov (ridge) regularization^77,78^ The resulting log-linear regression weights were applied to the edges (ROI to ROI correlations) of each network configuration within the four-state solution and the associated covariates. All results were cross validated.

Model development and validation consisted of four steps. First, model features were selected. Pearson correlation between each edge of the *k*^th^ (e.g., *k* = A^4^, B^4^, C^4^, and D^4^) dynamic brain state and clinical status was performed in the training set. Note that regarding dichotomous outcomes, a mass-univariate *t*-test would provide similar results as the Pearson correlation test concerning feature selection. Here, we used the Pearson correlation approach to be consistent with previous literature^27,52^. The resulting edges were separated into positive and negative groups, and thresholded on the basis of the statistical significance (*p* ≤ 0.05) and signs of correlation. Second, in the model development procedure, we first aggregated the values of edges in each feature set as a summary statistics, $S^k$, or “network strength”^27^ of the *k*^th^ brain state, for $k = 1,2,3,4$. The network strength and covariates (e.g., sex, age) were then entered into the model, yielding a scalar value, the predicated conditional probability of active psychotic symptoms. Formally, for subject *i*, $i \in \{ 1,2, \cdots ,n\}$, we define

$$\hat p_i^k: = {\Bbb P}\left( {Y_i = 1{\mathrm{|}}S^k = s_i^k,{\mathbf{C}} = {\mathbf{c}}_{\boldsymbol{i}}} \right) = \frac{{e^{\hat \beta _0^k + \hat \beta _1^ks_i^k + ({\hat{\boldsymbol \gamma }}^k) {\mathbf{c}}_{\boldsymbol{i}}}}}{{1 + e^{\hat \beta _0^k + \hat \beta _1^ks_i^k + ({\hat{\boldsymbol\gamma }}^k) {\mathbf{c}}_{\boldsymbol{i}}}}}$$

where $\hat p_i^k: = {\Bbb P}\left( {Y_i = 1{\mathrm{|}}S^k = s_i^k,{\mathbf{C}} = {\mathbf{c}}_{\boldsymbol{i}}} \right)$ denotes the predicted probability that subject *i* has active psychosis, given their observed network strength $s_i^k$ during brain state *k*, and covariates ${\mathbf{c}}_{\boldsymbol{i}}$, a vector consisting of all observed covariates for subject *i*. $\hat \beta _0^k,\hat \beta _1^k,$ and ${\hat{\boldsymbol \gamma }}^k$are estimated weights for brain state *k* from the elastic net logistic regression, where ${\hat{\boldsymbol \gamma }}^k$ is a vector consisting of the estimates for each covariate in ${\mathbf{c}}_{\boldsymbol{i}}$. Alternatively, we could model the predicted probability that subject *i* does not have psychosis as ${\Bbb P}\left( {Y_i = 0{\mathrm{|}}S^k = s_i^k,{\mathbf{C}} = {\mathbf{c}}_{\boldsymbol{i}}} \right) = \frac{1}{{1 + e^{\hat \beta _0^k + \hat \beta _1^ks_i^k + ({\hat{\mathbf \gamma }}^k)^\intercal {\mathbf{c}}_{\boldsymbol{i}}}}}.$ Probability estimation was iteratively performed using leave-one-subject-out cross-validation procedure. During each iteration, the weights were estimated using data from (n–1, *n* = 91) participants and were used to predict the probability of the remaining participant having active psychotic symptoms. Each individual was left out once; hence, the procedure yielded *n*-predicted probability scores. To evaluate the estimation performance, we measured the area under the receiver-operating characteristic (ROC) curve (AUC), estimated directly by conducting numerical integration of the ROC under all thresholds that yielded unique sensitivity/specificity values, wherein 0.5 indicates chance, and 1 is perfect discrimination.

The model development and parameter estimation were conducted only using the training data. To evaluate the reproducibility, we applied the model obtained from the training data, without further fitting or modification, to 39 previously unseen participants. To access the specificity of the model in detecting positive symptoms, we examined the positive, negative, and general psychopathology subscales of the PANSS in the held-out sample. PANSS scores were binarized as low (less than one standard deviation below the mean) or high (greater than one standard deviation below the mean) for each subscale (positive scale: 18.46 ± 6.51, cutoff = 11.95; negative scale: 13.48 ± 7.47, cutoff = 6.01; general psychopathology scale: 30.82 ± 7.95, cutoff = 22.86). Due to the large variance in PANSS negative scores, to thoroughly explore potential relations with this subscale, we additionally examined alternative thresholds of <15 and of <12 selecting the optimal result (<12) for comparison with the PANSS positive scale.

To assess the statistical significance of the sensitivity and specificity analyses, we performed nonparametric permutation testing. During each test, we first randomly permuted the active psychotic symptoms status or PANSS labels. Next, model fitting was carried out using the correct dynamic signatures and covariates and permuted (incorrect) labels. For each permutation, an AUC value was calculated. The permutation tests were performed 5000 times for the training and testing data, respectively. The null AUC values should be symmetrically distributed around 0.5 if the procedure is unbiased.

### Data availability

The data summary statistics that support the findings of this study are available from the corresponding author on request. As detailed in Holmes et al.^43^, the Brain Genomics Superstruct Project (GSP) initial release data set of structural, functional, and behavioral measures is available for download (http://neuroinformatics.harvard.edu/gsp/). Step-by-step instructions detailing how to access the release data set are available online in the “Request Access” page (http://neuroinformatics.harvard.edu/gsp/get). The patient data are not publicly available due to information that could compromise research participant privacy/consent.

## Electronic supplementary material

Supplementary Information(PDF 2547 kb)
