# Supplementary material for: The human cortex possesses a reconfigurable dynamic network architecture that is disrupted in psychosis
Source: Nat Commun. 2018 Mar 20;9:1157. doi: 10.1038/s41467-018-03462-y (PMC5861099; doi:10.1038/s41467-018-03462-y)
Supplement: Supplementary file 1 — Supplementary Information(PDF 2547 kb) [file 41467_2018_3462_MOESM1_ESM.pdf]

Supplementary information for "*The human cortex possesses a reconfigurable dynamic network architecture that is disrupted in psychotic illness*"

Jenna M. Reinen[1], Oliver Y. Chén[1], R. Matthew Hutchison[2], B. T. Thomas Yeo[3,4], Kevin M. Anderson[1], Mert R. Sabuncu[4,5], Dost Öngür[6], Joshua L. Roffman[7,8], Jordan W. Smoller[7], Justin T. Baker[6], Avram J. Holmes[1,4,7,9*]

[1]Department of Psychology, Yale University, New Haven, CT 06520

[2]Department of Psychology, Harvard University, Cambridge, MA 02138

[3]Department of Electrical & Computer Engineering, Clinical Imaging Research Centre, Singapore Institute for Neurotechnology & Memory Network Programme, National University of Singapore, 117583 Singapore

[4]Athinoula A. Martinos Center for Biomedical Imaging, Massachusetts General Hospital, Harvard Medical School, Charlestown, MA 02129

[5]School of Electrical and Computer Engineering and Meinig School of Biomedical Engineering, Cornell University, Ithaca, NY 14853

[6]Department of Psychiatry, Psychotic Disorders Division, McLean Hospital, Belmont, MA 02478

[7]Department of Psychiatry, Massachusetts General Hospital, Harvard Medical School, Boston, MA 02114

[8]Psychiatric Neuroimaging Research Division, Massachusetts General Hospital, Charlestown, MA 02129

[9]Department of Psychiatry, Yale University, New Haven, CT 06511

*Corresponding author.

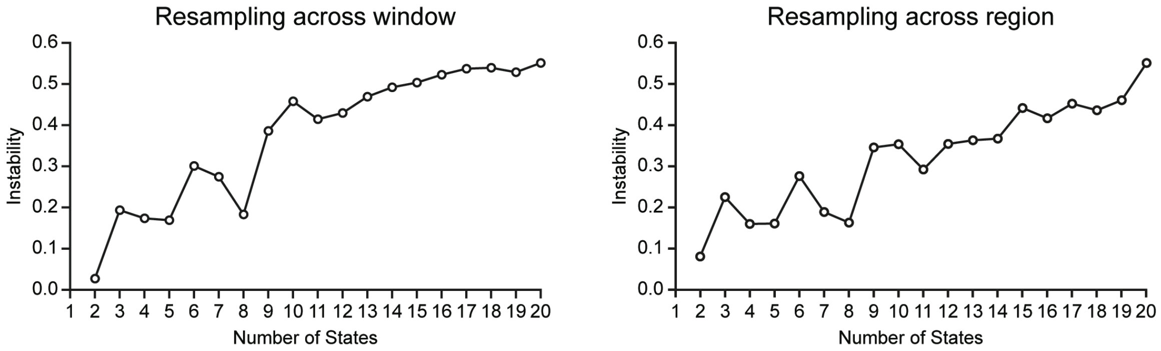

Supplementary Figure 1. Resampling across sliding windows and pairwise connectivity yield similar results. To examine the consistency of 2-20 possible state solutions, we employed a resampling method (1) to test the consistency across iterative clustering analyses. The results demonstrated relative stability at 2, 4, 5, and 8 state solutions across both approaches.

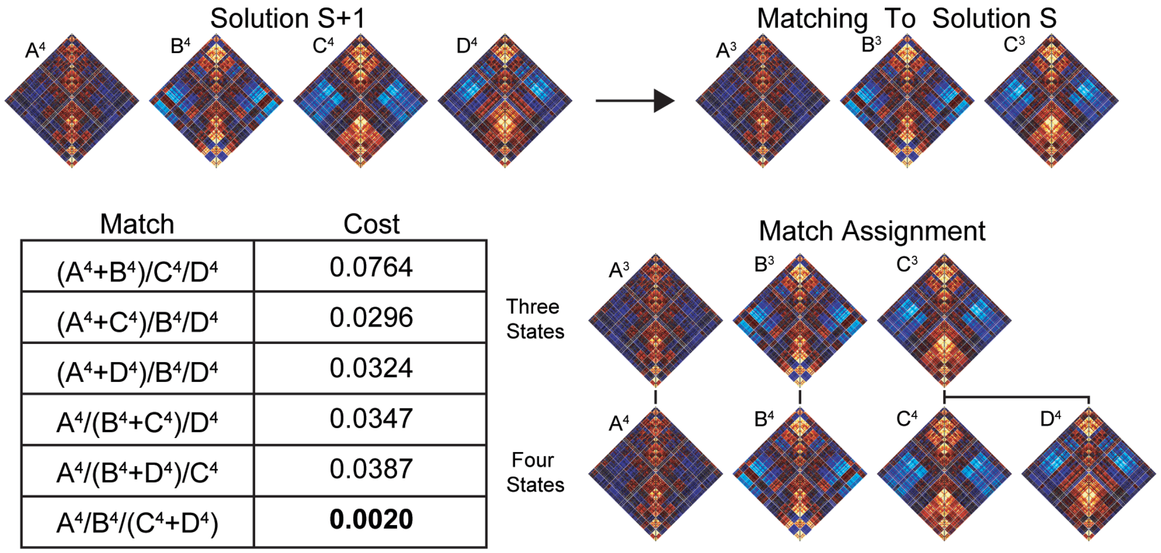

Match	Cost
$(A^4+B^4)/C^4/D^4$	0.0764
$(A^4+C^4)/B^4/D^4$	0.0296
$(A^4+D^4)/B^4/D^4$	0.0324
$A^4/(B^4+C^4)/D^4$	0.0347
$A^4/(B^4+D^4)/C^4$	0.0387
$A^4/B^4/(C^4+D^4)$	**0.0020**

Supplementary Figure 2. Hierarchy analysis across state solutions. To determine the relations linking brain states across clustering solutions, we used a Hungarian Matching technique to iteratively combine every state in state S + 1 (here, state solution 4) and match it to all states in state S (here, state solution 3). In this illustration, we found that a combination of states A^4/B^4 (C^4+D^4) in the four state solution best matched states $A^3/B^3/C^3$ in the three state solution because it had the lowest cost estimate. This process was repeated for state solutions 2-8. Results are then represented graphically in Figure 2 within the main text.

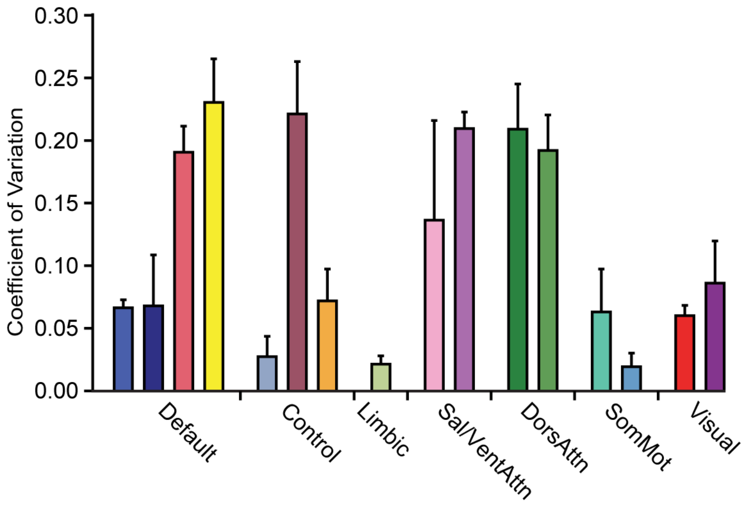

Supplementary Figure 3. Variation across networks across state solutions 2-8. The bar graph represents the magnitude of the coefficient of variation for each sub-network across all state solutions. Greater values of variability are associated with default, control, and attention networks relative to limbic, somato-motor and visual networks.

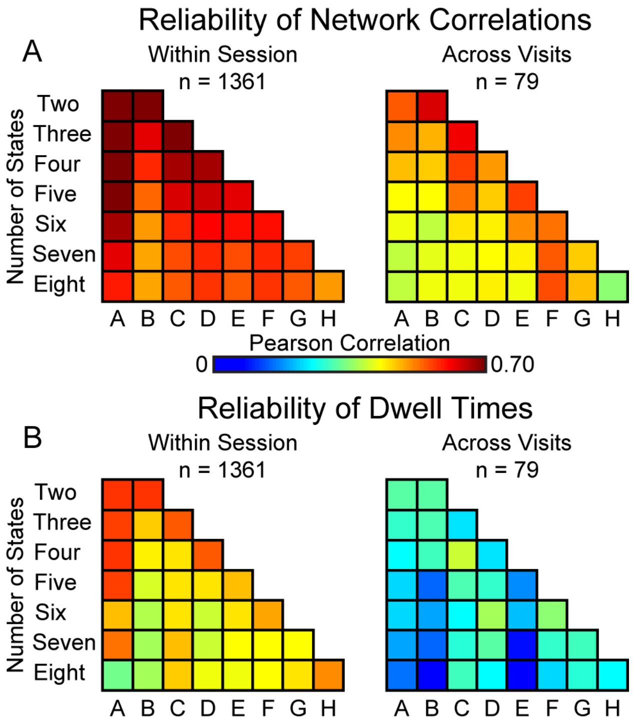

Supplementary Figure 4. Brain states and dwell times are reliable within and across visits. (A) Within-participant Pearson correlations between bold runs one and two (n=1361) were calculated for each state across all ROIs. Results indicated a significant level of consistency in within-individual connectivity profiles across same-day bold sessions, as well as across visits (n=79) within six months of each other. (B) Likewise, dwell time correlations showed consistency within participants across sessions and visits.

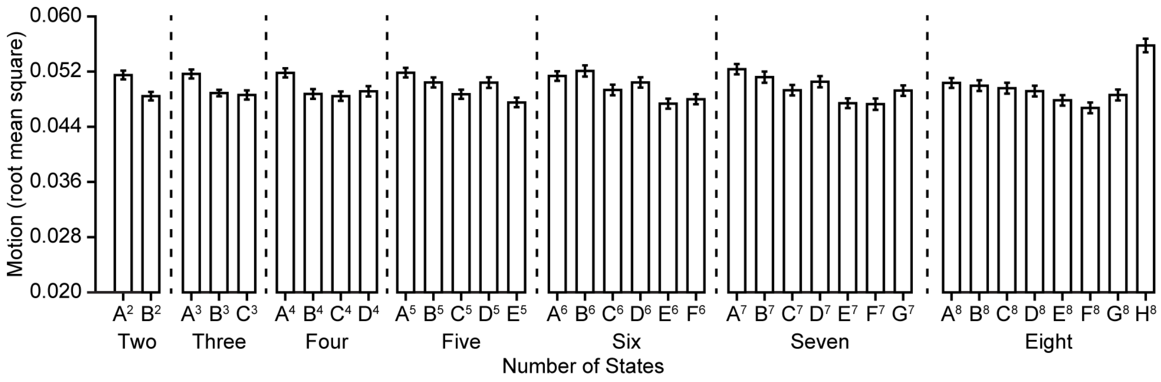

Supplementary Figure 5. Root mean square motion across brain states. State A associates with heightened motion for solutions 2 through 4 and shows a muted increase in motion in solutions 5 through 7.

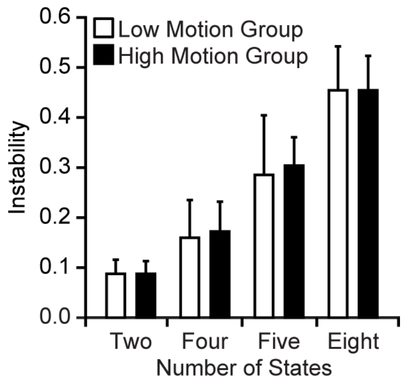

Supplementary Figure 6. Consistent clustering solution stability was observed across high and low motion participants. Mean clustering instability and standard deviation is presented for groups defined by their relative motion estimates.

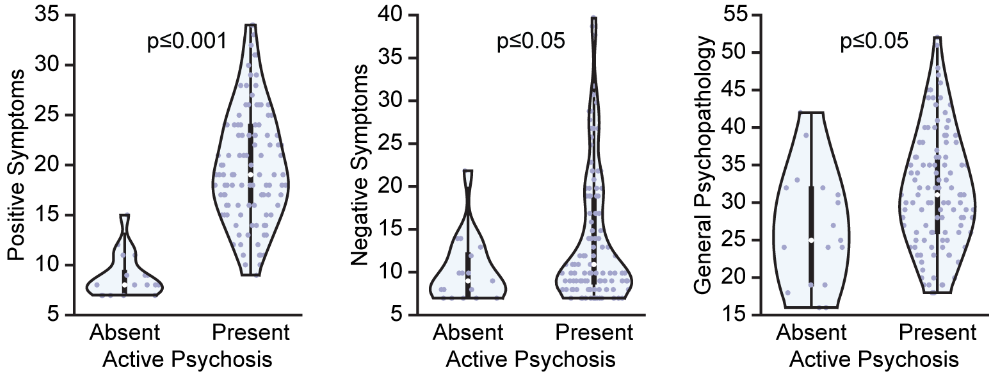

Supplementary Figure 7. Violin plots of Positive and Negative Syndrome Scale (PANSS) scores (positive, negative, and general psychopathology scales) for the active psychosis groups. The absence or presence of active psychosis is based on the DSM-IV (SCID) clinician rated presence of delusions and/or hallucinations in the past month.

Supplementary Table 1. Mean within network connectivity for patients with psychosis and the matched comparison participants

	State A[4]				State C[4]			
Network	Comparison	Patients	T-Statistic	P value	Comparison	Patients	T-Statistic	P value
Control A	0.30±0.006	0.27±0.010	2.15	0.03	0.27±0.006	0.23±0.010	3.15	0.01
Control B	0.34±0.006	0.28±0.010	5.95	0.001*	0.52±0.008	0.45±0.014	4.85	0.001*
Control C	0.67±0.010	0.60±0.015	4.12	0.001*	0.68±0.010	0.62±0.019	2.49	0.01
Default A	0.39±0.006	0.37±0.011	1.69	0.09	0.62±0.009	0.59±0.016	1.31	0.19
Default B	0.38±0.006	0.31±0.010	6.08	0.001*	0.51±0.009	0.46±0.016	2.91	0.01
Default C	0.44±0.009	0.44±0.015	0.21	0.83	0.44±0.011	0.41±0.014	1.38	0.17
Default D	0.70±0.020	0.66±0.022	1.50	0.13	0.69±0.020	0.65±0.033	1.24	0.22

Supplementary Table 1. Values reflect mean within network connectivity for patients with psychosis and the matched comparison participants ± Standard Error. Network connectivity was examined in the four state solution. Following correction for nuisance variables, t-tests were used to make direct comparisons in connectivity magnitude between the two groups. Differences in degrees of freedom reflect the number of participants who expressed a given state. * Denotes comparisons that pass multiple comparison correction (Bonferroni p≤0.05).
